# P-1535. Omadacycline was Shown to Preserve the Microbiome in a Murine Model of Post-Influenza MRSA Pneumonia

**DOI:** 10.1093/ofid/ofaf695.1716

**Published:** 2026-01-11

**Authors:** Sumiko Gomi, Emily Price, Sabrina Faozia, Jessica V Pierce, Sarah E Hobdey

**Affiliations:** Idaho Veterans Research and Education Foundation; Boise VA Medical Center, Boise, Idaho; Idaho Veterans Research and Education Foundation; Boise VA Medical Center, Boise, Idaho; Idaho Veterans Research and Education Foundation; Boise VA Medical Center, Boise, Idaho; Paratek Pharmaceuticals, Inc., Medford, MA; Idaho Veterans Research and Education Foundation; Boise VA Medical Center; Idaho State University, Boise, Idaho

## Abstract

**Background:**

Secondary bacterial pneumonia caused by methicillin resistant *Staphylococcus aureus* (MRSA) is a leading cause of death following influenza A virus (IAV) infection. Gut dysbiosis is a major contributor to bacterial superinfection due to changes in pulmonary immunity from decreased short-chain fatty acid (SCFA) production. Omadacycline is an FDA-approved antibiotic for the treatment of adult community acquired bacterial pneumonia. Omadacycline improved survival in a murine model of post-IAV-MRSA pneumonia, but the impact on the microbiome in this model is unknown. This study assessed the effect of omadacycline on the microbiome in a murine model of IAV-MRSA pneumonia.

**Figure 1.**
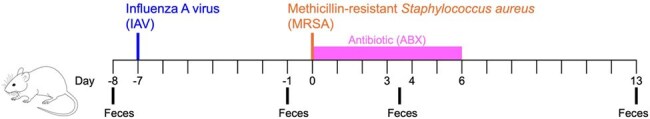
Murine Model of Post-Influenza Methicillin-Resistant S. aureus Infection Female BALB/c mice were infected intranasally with influenza A/Puerto Rico/8/34 (H1N1) virus, and 7 days post-infection with CA-MRSA USA300. Antibiotics (omadacycline (intraperitoneally), linezolid (orally) or vehicle were administered twice daily for six days. Feces were collected on the appropriate days for 16S rRNA gene profiling and SCFA quantification.

**Figure 2.**
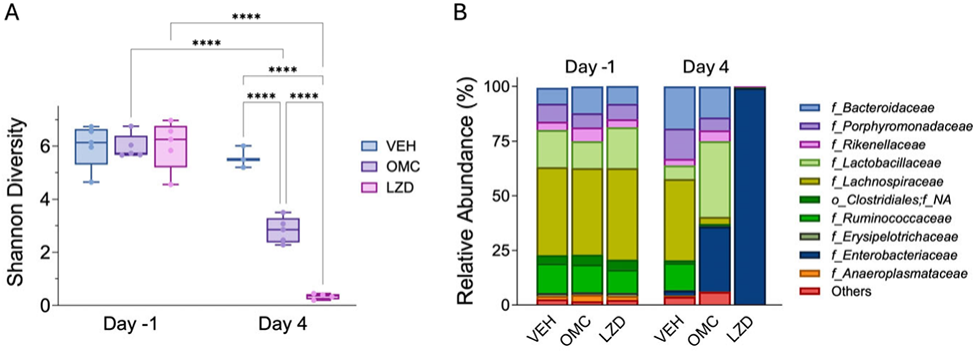
Omadacycline Preserves the Alpha Diversity and Composition in the Gut Microbiota in IAV-MRSA Infected Antibiotic-Treated Mice in Comparison to Linezolid (A) Alpha diversity (Shannon index) of fecal microbiota in control (VEH), omadacycline (OMC), and linezolid (LZD) groups, as assessed by 16S rRNA gene sequencing. A reduction in diversity was observed in the OMC group and diversity was highly reduced in the LZD group compared to the control group at Day 4. (B) Relative abundance of bacterial taxa at the family level. Antibiotic treatment induced distinct compositional shifts, with an increase in Enterobacteriaceae observed in both OMC and LZD groups, while Enterobacteriaceae was the dominant family observed in the LZD group. IAV=influenza A virus, MRSA=methicillin-resistance Staphylococcus aureus.

**Methods:**

Mice infected with IAV and MRSA were treated with omadacycline or linezolid (Fig. 1). Fecal samples were collected for 16S rRNA sequencing and analyzed by LC-MS for the quantification of SCFA.

**Results:**

Alpha diversity decreased during antibiotic treatment, but alpha diversity of the linezolid treatment groups was lower than the omadacycline treatment groups. More specifically, during omadacycline treatment, there was a decrease in *Lachnospiraceae* and *Ruminococcacae* and an increase in *Lactobacillaceae* and *Bacteroidaceae* (Fig. 2) while during linezolid treatment, there was an expansion of *Enterobacteriaceae*. After treatment, the diversity of the omadacycline treatment groups more closely reflected the initial microbiome than the linezolid treatment groups. SCFAs decreased during treatment and recovered post-treatment in all groups. The reduction of acetic and propionic acid due to linezolid treatment was greater than that of omadacycline treatment. Omadacycline treatment resulted in a faster recovery and improved survival in comparison to linezolid in IAV-MRSA infected mice.

**Conclusion:**

Treatment with omadacycline maintained a higher level of diversity in the gut microbiome along with increased levels of SCFAs compared to linezolid and promoted diversity recovery along with improved survival. These results suggest omadacycline may preserve diversity in the gut microbiome compared to other antibiotics, leading to improved recovery during IAV-MRSA pneumonia, and further study is warranted.

**Disclosures:**

Jessica V. Pierce, PhD, Paratek Pharmaceuticals, Inc.: Employee

